# Surgical Anatomy of the Microscopic and Endoscopic Transorbital Approach to the Middle Fossa and Cavernous Sinus: Anatomo-Radiological Study with Clinical Applications

**DOI:** 10.3390/cancers15184435

**Published:** 2023-09-06

**Authors:** Simona Serioli, Mariagrazia Nizzola, Pedro Plou, Alessandro De Bonis, Jenna Meyer, Luciano C. P. C. Leonel, Andrea A. Tooley, Lilly H. Wagner, Elizabeth A. Bradley, Jamie J. Van Gompel, Maria Elena Benini, Iacopo Dallan, Maria Peris-Celda

**Affiliations:** 1 Division of Neurosurgery, Department of Medical and Surgical Specialties, Radiological Sciences and Public Health, University of Brescia, 25123 Brescia, Italy; s.serioli002@unibs.it; 2 Rhoton Neurosurgery and Otolaryngology Surgical Anatomy Program, Mayo Clinic, Rochester, MN 55905, USA; nizzola.mariagrazia@hsr.it (M.N.); pedro.plou@hospitalitaliano.org.ar (P.P.); debonis.alessandro@hsr.it (A.D.B.); meyer.jenna@mayo.edu (J.M.); leonel.luciano@mayo.edu (L.C.P.C.L.); 3 Department of Neurologic Surgery, Mayo Clinic, Rochester, MN 55905, USA; vangompel.jamie@mayo.edu; 4 Department of Neurosurgery and Gamma Knife Radiosurgery, I.R.C.C.S. San Raffaele Scientific Institute, Vita-Salute University, 20132 Milan, Italy; 5 Neurosurgery Department, Hospital Italiano de Buenos Aires, Buenos Aires C1199, Argentina; 6 Department of Neurologic Surgery, Mayo Clinic, Phoenix, AZ 85054, USA; 7 Department of Ophthalmology, Mayo Clinic, Rochester, MN 55905, USA; tooley.andrea@mayo.edu (A.A.T.); wagner.lilly@mayo.edu (L.H.W.); bradley.elizabeth@mayo.edu (E.A.B.); 8 Department of Otolaryngology/Head and Neck Surgery, Mayo Clinic, Rochester, MN 55905, USA; 9 Department of Neurosurgery—Head and Neck Surgery, Azienda Ospedaliero-Universitaria Pisana, 56126 Pisa, Italy; mariaelena.benini@hotmail.it; 10 Department of Otolaryngology—Head and Neck Surgery, Azienda Ospedaliero-Universitaria Pisana, 56126 Pisa, Italy; iacopo.dallan@gmail.com

**Keywords:** anatomy, anterior cranial fossa, endoscopy, microscope, middle cranial fossa, skull base, transorbital approach

## Abstract

**Simple Summary:**

The popularity that transorbital approaches (TOAs) gained in recent years has allowed their diffusion into skull base surgery. However, for their correct application, it is essential to know the potential and the limits of this group of transorbital routes, as well as the knowledge of anatomy from this relatively new surgical perspective. In this paper, the authors illustrate step-by-step the superior eyelid transorbital approach from a macroscopic and endoscopic perspective, highlighting the main anatomical relationships to understand the application of this surgical route for the treatment of skull base pathologies. Surgical cases are provided to illustrate indications for this approach.

**Abstract:**

Background: The transorbital approaches (TOAs) have acquired growing notoriety, thanks to their ability to offer alternative corridors to the skull base. However, the limited access and the unfamiliarity with this surgical perspective make recognition of key landmarks difficult, especially for less experienced surgeons. The study wants to offer a detailed description of the anatomy to comprehend the potential and limitations of TOAs. Methods: Measurements of the orbit region and the surrounding areas were performed on two hundred high-resolution CT scans and thirty-nine dry skulls. Five specimens were dissected to illustrate the TOA, and one was used to perform the extradural clinoidectomy. Three clinical cases highlighted the surgical applications. Results: A step-by-step description of the key steps of the TOA was proposed and a comparison with the transcranial anterior clinoidectomy was discussed. The mean work distance was 6.1 ± 0.4 cm, and the lateral working angle increased 20 ± 5.4° after removing the lateral orbital rim. Conclusions: TOAs are indicated in selected cases when tumor involves the lateral portion of the cavernous sinus or the middle skull base, obtaining a direct decompression of the optic nerve and avoiding excessive manipulation of the neurovascular structures. Comprehension of surgical anatomy of the orbit and its surrounding structures is essential to safely perform these approaches.

## 1. Introduction

The evolution of neurosurgical techniques that occurred in the last 20 years has favored the development of increasingly minimally invasive approaches for the treatment of skull base pathologies [1,2,3]. The utilization of the endoscope and its technological development has progressively revolutionized skull base surgery thanks to the improvement in imaging (4K ultra-high-definition endoscope), surgical instrumentations, and the introduction of fluorescent dyes [4,5,6]. The endonasal (EEAs) and transorbital approaches (TOAs) have acquired more and more notoriety, both in support and in replacement of the classic transcranial approaches for selected cases [7,8,9,10].

In 2010, the term “transorbital neuroendoscopic surgery” (TONES) was created for the first time to define a group of endoscopic approaches that offered alternative surgical corridors for the treatment of skull base pathologies [8].

The application of TOAs has been subsequently extended to the lesions of the anterolateral skull base such as meningiomas, schwannomas, dermoid cysts, CSF leak, and infections [8,11,12,13,14,15,16,17,18,19,20,21,22,23,24], using the orbital cavity as a corridor to offer several trajectories [8,13,24,25,26,27,28,29,30,31,32,33,34,35,36,37,38,39,40]. Moreover, recent anatomical and initial clinical studies have also highlighted the potential versatility of these approaches, to the tentorial area [41,42], petrous apex [43,44,45], and the insular region [46,47].

However, the limited surgical view and workspace, and the unfamiliarity with the surgical perspective offered by TOAs make recognition of key surgical landmarks challenging, especially for less experienced surgeons. This study offers a detailed step-by-step description of the superior eyelid transorbital route, identifying the main anatomical landmarks to better understand the principles and applicability of this innovative surgical approach. The microscopic and endoscopic view of the main passages is offered to better understand the anatomical relationships. Moreover, the anatomy of the orbital cavity and the adjacent region have been studied, highlighting the most important applications of this approach.

## 2. Materials and Methods

The research was reviewed by the Institutional Review Board and conducted according to the ethical guidelines of the Declaration of Helsinki. The patients’ consent was obtained.

### 2.1. Morphometric Study on Dry Skulls

**Figure 1:**
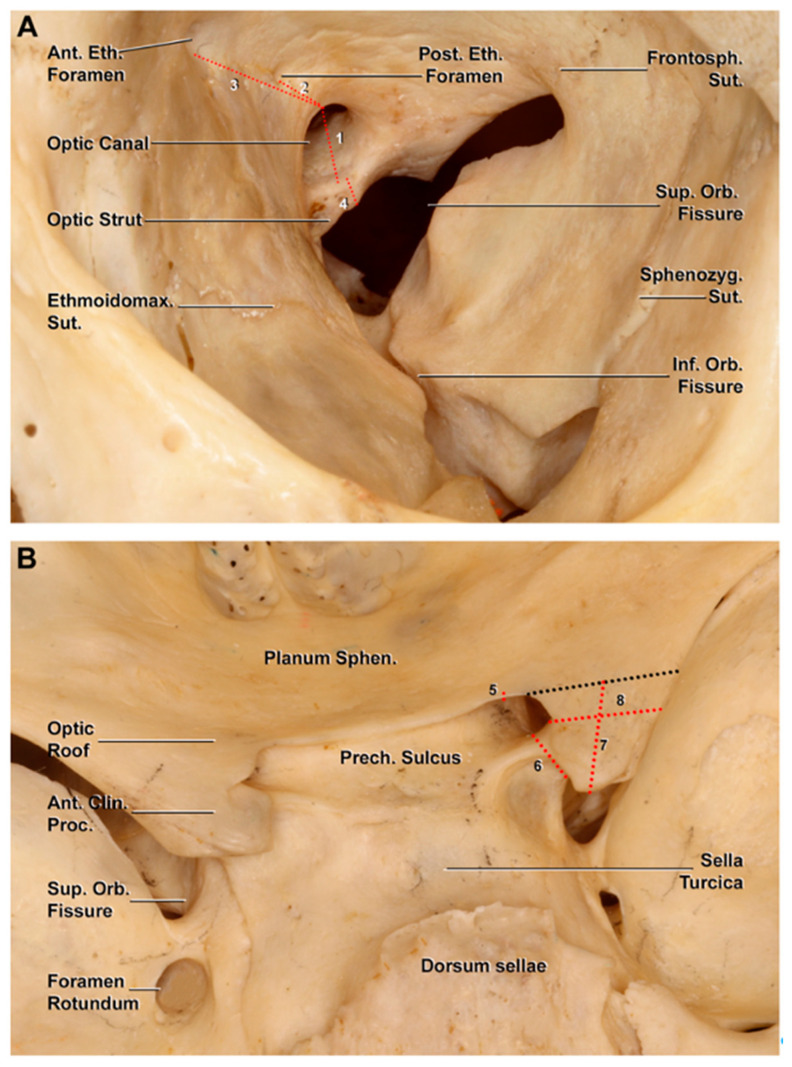
Bony anatomy of the orbital cavity and middle cranial fossa on a dry skull. The measurements are indicated with a dotted red line. (**A**) Osseous landmarks of the orbit region: 1, height of the optic canal; 2, distance between the optic canal and posterior ethmoidal foramen; 3, distance between the optic canal and anterior ethmoidal foramen; 4, optic strut thickness. (**B**) Superior view of the middle cranial fossa. The dotted black line represents the projection of the anterior limit of the anterior clinoid process; 5, height of the optic roof; 6, optic strut thickness (endocranial view); 7, length along the longitudinal axis of the anterior clinoid process; 8, width of the anterior clinoid process. Ant. Clin. Proc., anterior clinoid process; Ant. Eth. Foramen, anterior ethmoidal foramen; Ethmoidomax. Sut., ethmoidomaxillary suture; Frontosph. Sut., frontosphenoidal suture; Inf. Orb. Fissure, inferior orbital fissure; Planum Sphen., planum sphenoidale; Post. Eth. Foramen, posterior ethmoidal foramen; Prech. Sulcus, prechiasmatic sulcus; Sphenozyg. Sut., sphenozygomatic suture; Sup. Orb. Fissure, superior orbital fissure.

Thirty-nine dry skulls (78 sides) were studied by two authors (S.S. and M.N.) to analyze the main bony relationships between the orbit region and middle cranial fossa. A total of eight measurements were performed with the use of a sliding caliper (Figure 1): four measurements were performed from an endocranial perspective (optic roof, optic strut, maximum length, and width of the anterior clinoid process (ACP)), while the remaining ones (optic foramen, optic strut, the distance between the medial border of the optic foramen and anterior and posterior ethmoidal canals) were performed at the level of the orbital cavity (Figure 1).

### 2.2. Radiological Study

Two hundred high-resolution CT scans (400 sides) were utilized to measure the ACP length, optic roof, and optic strut, and the working distance to the tip of the ACP from the orbital floor.

The course of the optic canal and the ACP pneumatization was classified according to DeLano’s classification [48] into 4 different groups:

- Type 1: the optic canal was adjacent to the sphenoid sinus (SpS) without impression;
- Type 2: the optic canal caused an impression on the SpS;
- Type 3: the optic canal was identified in the SpS;
- Type 4: the optic canal was found lateral to the SpS and the posterior ethmoid sinus (presence of Onodi cell or spheno-ethmoidal air cell).

The measurement of the working angle on the axial plane was evaluated on five post-op CT scans in patients who underwent a TOA. Considering the tangent to the lateral orbital rim as the lateral limit of the surgical corridor, we measured the additional working angle on the axial plane in these cases in which the frontal process of the zygomatic bone was removed.

The radiological measurements for the morphological analysis were obtained through a dedicated analysis image software (QREADS 5.15.0, Copyright 2023, Mayo Clinic).

### 2.3. Anatomical Dissections

Ten sides of five embalmed latex-injected human heads were dissected to illustrate the main surgical steps of the TOA. The dissection included a first phase of soft tissue exposure (superior eyelid incision, orbicularis oculi muscle division, dissection of periosteal layer over the frontal process of the zygomatic bone, and osteotomy of the lateral orbital rim), followed by the endoscopic/microscopic phase. A rigid endoscope (4 mm in diameter, and 18 cm in length), paired with 0°, 30°, or 45° rod lenses (Stryker Corp, Kalamazoo, MN, USA), was used.

A standard pterional approach with subsequent extradural anterior clinoidectomy was performed on an embalmed latex-injected specimen for the comparison of the two surgical corridors to the ACP.

The statistical evaluation was performed with Jamovi Version 2 statistic software (The jamovi project (2022) (Version 2.3) [Computer Software] https://www.jamovi.org, accessed on 1 February 2023), and the final values were expressed as percent, mean, and standard deviation.

## 3. Results

### 3.1. Step-by-Step Dissection

**Figure 2:**
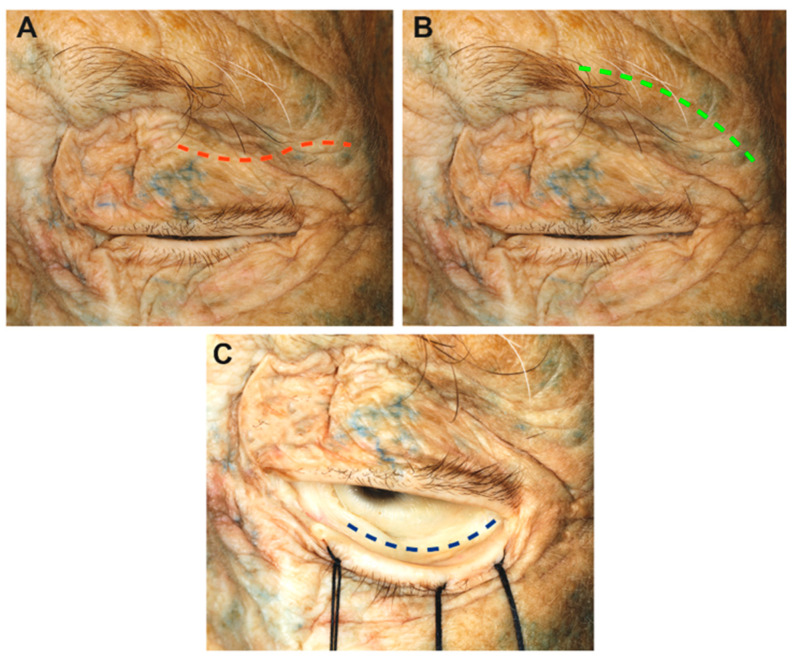
Summary of the different skin incisions: (**A**) transpapebral (red dotted line), (**B**) supraorbital transciliary (green dotted line), (**C**) inferior eyelid conjunctival incision (blue dotted line).

The specimen was positioned supine with the head in a neutral position, and a 3 cm S-shaped superior eyelid skin incision was made 5 mm above the upper lid lash line following the skinfold (Figure 2).

**Figure 3:**
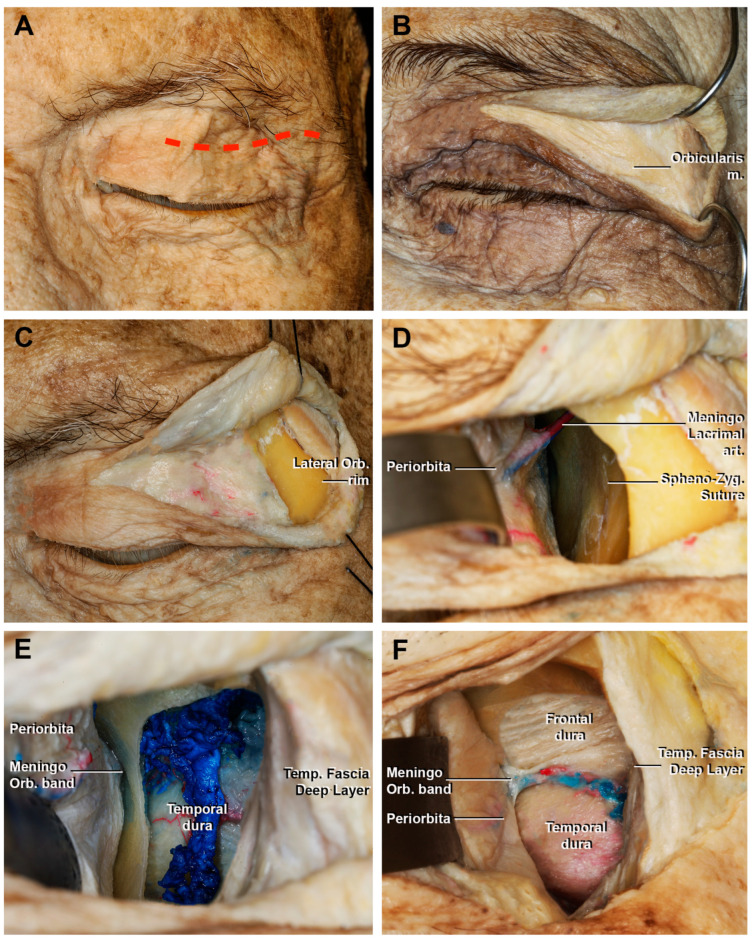
Macroscopical view of the superior eyelid transorbital endoscopic with the removal of the lateral orbital rim. (**A**) Skin incision (red dotted line); (**B**) detail of fibers that form the palpebral portion of the orbicularis muscle; (**C**) incision of the orbicularis muscle and exposure of the lateral orbital rim. (**D**) Sub-periorbital dissection. The sphenozygomatic suture and the meningo-lacrimal artery are exposed. (**E**) After the craniectomy and, in this case, the removal of the lateral orbital rim, the temporal dura and the temporal muscle are exposed; (**F**) extension of the craniectomy superiorly, with the identification of the frontal dura mater. The meningo-orbital band, which connects the periorbita with the fronto-temporal dura, is shown. Lateral Orb. rim, lateral orbital rim; Meningo Lacrimal art., meningo lacrimal artery; Meningo Orb. band, meningo-orbital Band; Temp. Fascia Deep Layer, temporal fascia deep layer.

The palpebral portion of the orbicularis ocularis muscle was exposed (Figure 3A). The incision extended through the anterior lamella, avoiding premature entering of the orbital compartment delineated by the orbital septum, the lacrimal gland, the anterior surface of the tarsus, and the tendon of the levator palpebrae muscle [31,38,39]. Dissection was continued in a sub-orbicularis oculi plane to the lateral orbital rim.

If a wider surgical corridor was required, the incision was further extended laterally to directly expose the margin of the lateral orbital rim (LOR) after its identification by palpation (Figure 3B,C).

Once exposed, the periosteum was divided at the level of the frontal process of the zygomatic bone and subperiorbital dissection was performed (Figure 3D). During this procedure, it was important to protect the periorbita. The LOR was removed after detaching the temporalis muscle to obtain a larger surgical corridor and greater maneuverability.

The sphenozygomatic suture was exposed up to the meningo-orbital foramen (when it is present), where the meningo-lacrimal artery, an orbital branch of the middle meningeal artery, runs. This artery could be used as a landmark to identify the proximity to the meningo-orbital band and the superior orbital fissure (SOF). During the dissections, the foramen and the meningo-lacrimal artery were recognized in eight cases (80%).

From this point, the use of the malleable retractor was required. The spheno-frontal suture was considered the projection of the Sylvian fissure [34,49]. Once the meningo-lacrimal artery was divided, the most lateral portion of the superior orbital fissure could be identified. Inferiorly, the zygomaticofacial nerve and its homonymous artery identified the lateral border of the inferior orbital fissure (IOF).

#### 3.1.1. Craniectomy

**Figure 4:**
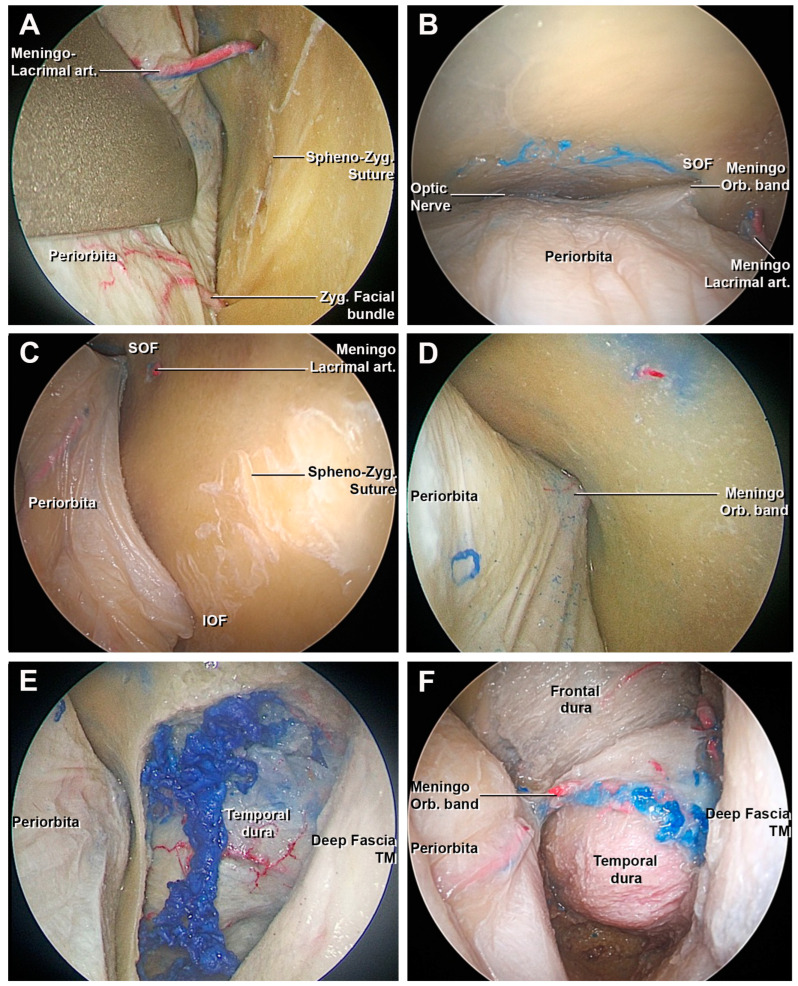
Endoscopic view of the superior eyelid transorbital endoscopic, intraorbital step. (**A**) Sub-periorbital dissection with the exposure of the sphenozygomatic suture and the meningo-lacrimal artery. (**B**) View from above of the periorbita. From medial to lateral: the optic nerve, superior orbital fissure, meningo-orbital band, and meningo-lacrimal artery. (**C**) After cutting of the meningo-lacrimal artery, the area of the craniectomy is delimited medially by the lateral border of the SOF and IOF. The meningo-orbital band is finally identified. (**D**) Detail of the meningo-orbital band in the SOF. (**E**) After the craniectomy, the temporal dural and the temporal muscle are identified. (**F**) Cranial extension of the craniectomy in order to expose the frontal dural and the meningo-orbital band. IOF, inferior orbital fissure; Meningo-Orbital band, meningo-orbital band; Meningo-lacrimal art., meningo-lacrimal artery; Spheno-Zyg. Suture, spheno-zygomatic suture; SOF, superior orbital fissure; TM, temporal muscle; Zyg. Facial bundle, zygomatic facial bundle.

The craniectomy was performed by drilling the orbital surface of the body of the zygomatic bone and the greater wing of sphenoid bone (GWS), using the SOF and IOF as the medial border, and the frontozygomatic suture as the superior limit. After this step, the temporalis muscle and the dura of the temporal pole were exposed (Figure 3E,F). If an extension of the approach to the frontal lobe was required, the lesser wing of the sphenoid bone (LWS) and the orbital portion of the frontal bone could be drilled to amplify the craniectomy (Figure 4). The remaining bony egg sheet was removed with a dissector, and the dura acted as a barrier to potential injury of the intradural structures. At the end of the drilling the residual medial portion of the GWS, a triangular bone spicule defined the sagittal crest (Figure 4E) [50] and was subsequently removed to expose the meningo-orbital band and the transition from periorbita to lateral wall of cavernous sinus and the medial portion of the temporal dura [40].

#### 3.1.2. Access to the Middle Fossa and Petrous Apex

**Figure 5:**
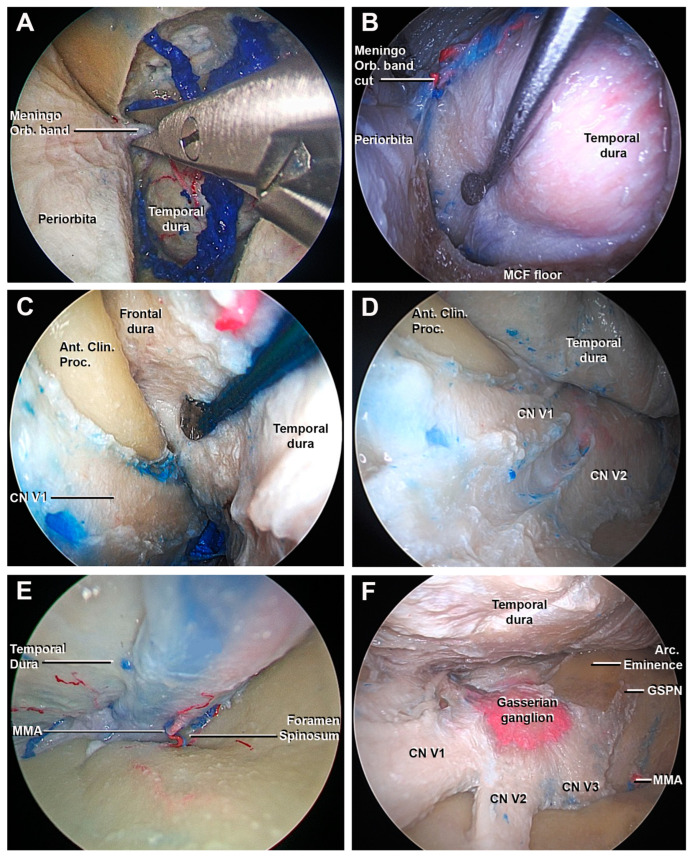
Endoscopic perspective of the superior eyelid transorbital endoscopic after the craniectomy: endocranial step. (**A**) The cutting of the meningo-orbital band using a microscissor is performed. (**B**–**D**) The interdural dissection between the meningeal layer of the lateral wall of the cavernous sinus and the temporal dura is obtained using a surgical route from lateral to medial: the lateral surface of anterior clinoid process and the cavernous sinus are shown. (**E**) The middle meningeal artery is found at the level of the foramen spinosum; after the cutting, the extradural peeling of the middle skull base is completed. (**F**) Final view of the middle cranial fossa: the gasserian ganglion, the three branches (V1, V2, and V3) of the trigeminal nerve, and the GSPN are identified. Ant. Clin. Proc., anterior clinoid process; Arc. Eminence, arcuate eminence; GSPN, greater superficial petrosal nerve; MMA, middle meningeal artery; MCF, middle cranial fossa; Meningo-Orbital band, meningo-orbital band; V1, ophthalmic division of CN V; V2, maxillary division of CN V; V3, mandibular division of CN V.

The MOB is a dural band that connects the periorbita with the fronto-temporal dura (Figure 4D) [40]. The division of the MOB was a necessary step to proceed safely with the interdural peeling of the cavernous sinus (CS) and middle skull base (Figure 5A).

**Figure 6:**
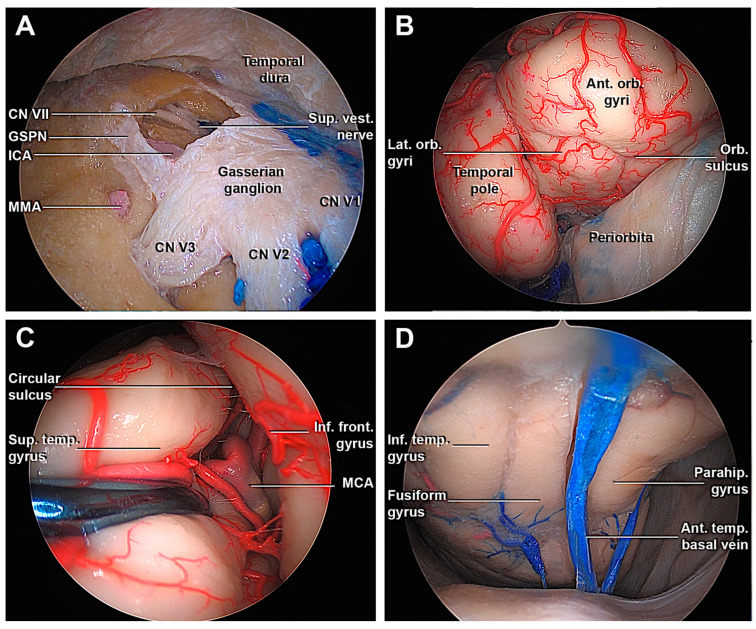
Transorbital endoscopic approach to the middle fossa. (**A**) Drilling through the Kawase’s rhomboid, the petrosal tract of ICA, the facial nerve, and the superior vestibular nerve are exposed. (**B**) The opening of the fronto-temporal dura from an endoscopic transorbital perspective shows the temporal pole and infero-medial portion of the frontal lobe. (**C**) Following the circular sulcus, it is possible to identify the passages between the M2-M3 segment of the MCA. (**D**) Detail of the inferior surface of the temporal lobe and its drainage. Ant. Orb. Gyri, anterior orbital gyri; CN, cranial nerve; GSPN, greater superficial petrosal nerve; ICA, internal carotid artery; Inf. Front. Gyrus, inferior frontal gyrus; Inf. temp. Gyrus, inferior temporal gyrus; MMA, middle meningeal artery; Orb. Sulcus, orbital sulcus; Parahip. Gyrus, parahippocampal gyrus; Sup. Temp. gyrus, superior temporal gyrus; Sup. Vest. Nerve, Superior vestibular nerve; V1, ophthalmic division of CN V; V2, maxillary division of CN V; V3, mandibular division of CN V.

Starting from the SOF, an interdural dissection was performed to separate the temporal dura from the meningeal layer of the lateral wall of the cavernous sinus (LWCS). The direction of the dissection was parallel to the cranial nerves of the LWCS to avoid damaging them or to accidentally enter in the CS, injuring the cavernous segment of the internal carotid artery (ICA) (Figure 5B–D). The extradural peeling at the level of MCF could be difficult if bony prominences of the greater sphenoid wing’s floor have not been adequately smoothened, for example, due to the presence of a midsubtemporal ridge [33,41]. To complete temporal dural detachment, the middle meningeal artery (MMA) was divided at the level of the foramen spinosum (Figure 5E). Next, the exposure of the gasserian ganglion, the petrous apex, and arcuate eminence were achieved (Figure 5F). Anterior petrosectomy at the level of the Kawase rhomboid area was performed (Figure 6A), revealing the petrosal tract of the ICA, the facial nerve, and the superior vestibular nerve.

#### 3.1.3. Access to the Lateral Wall of the Cavernous Sinus and Anterior Clinoidectomy

**Figure 7:**
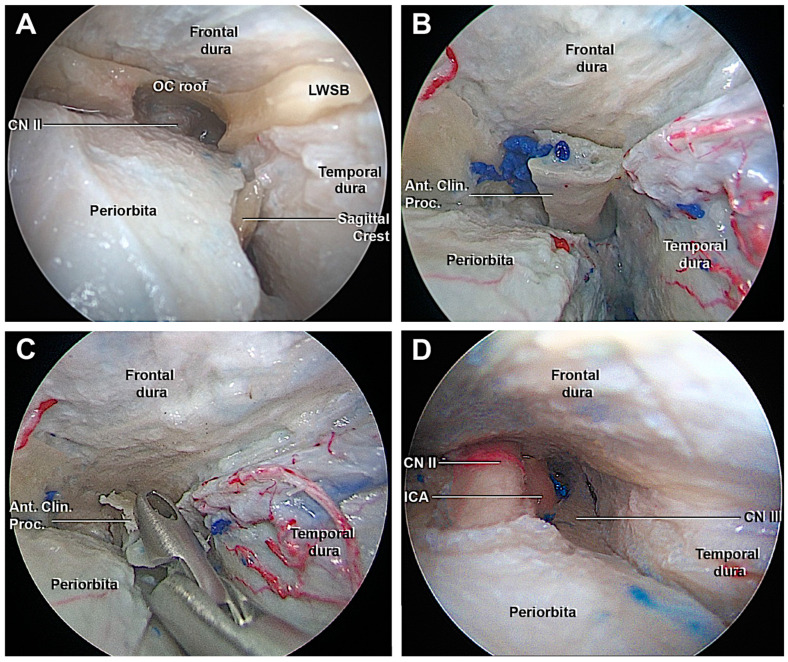
Endoscopic view of the transorbital extradural anterior clinoidectomy. (**A**) After the drilling of the orbital surface of the body of the zygomatic bone and the greater sphenoid wing, the optic roof, optic strut, and the lesser sphenoid wing anchor the anterior clinoid process. The roof of the orbital canal is opened to expose the CN II. (**B**) The interdural dissection of the lateral wall of the cavernous sinus allows to detach the anterior clinoid process from the surrounding dura and facilitate its removal. (**C**) Removal of the ACP. (**D**) Final view after the transorbital extradural anterior clinoidectomy. From medial to lateral: the optic nerve, ICA, and third cranial nerve, which are delimited superiorly by the frontal dura, laterally by the temporal dura. Ant. Clin. Proc., anterior clinoid process; CN II, optic nerve, CN III, oculomotor nerve; ICA, internal carotid artery; LWSB, lesser wing sphenoid bone.

**Figure 8:**
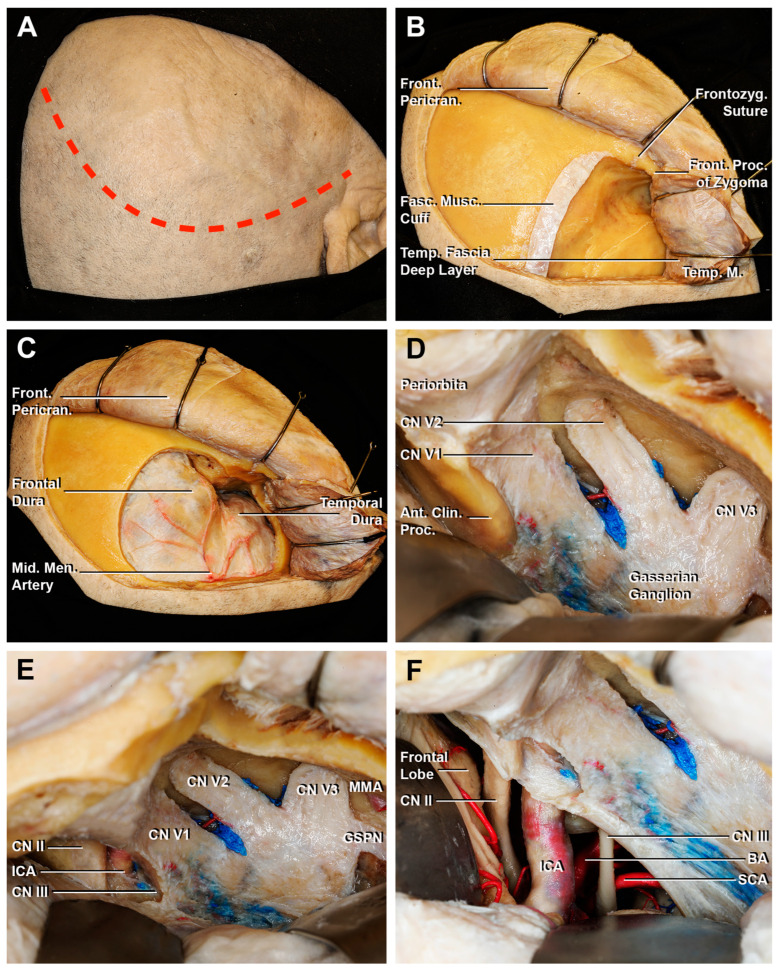
Key steps of an extradural anterior clinoidectomy through a fronto-temporal craniotomy. (**A**) A curvilinear skin incision is performed (red dotted line). (**B**) The temporal muscle is cut, creating the musculo-fascial cuff. Then, the muscle is completely retracted in order to expose the surface for the bone flap. (**C**) After the fronto-temporal craniotomy, the fronto-temporal dura is exposed. The course of the anterior branch of the middle meningeal artery is shown. (**D**) The middle fossa dura is elevated until the superior orbital fissure, which exposes the meningo-orbital band. After the cutting of the meningo-orbital band at the level of the sphenoid ridge, the peeling of the dura propria is completed until exposing the bony surface of the anterior clinoid process. (**E**) After the drilling of the anterior clinoid process, the paraclinoid space is exposed: the clinoid segment of ICA, CN II, and CN III are identified. (**F**) The opening of the dura allows an intradural surgical view. Ant. Clin. Proc., anterior clinoid process; BA, basilar artery; CN II, optic nerve; CN III, oculomotor nerve; Front. Pericran., frontal pericranium; Front. Proc. of Zygoma, frontal process of the zygoma; Frontozyg. Suture, frontozygomatic suture; Fasc. Musc. Cuff., fascial muscular cuff; ICA, internal carotid artery; GSPN, greater superficial petrosal nerve; Mid. Men. Artery, middle meningeal artery; SCA, superior cerebellar artery; Temp. Fascia Deep Layer, temporal fascia deep layer; Temp. M., temporal muscle; V1, ophthalmic division of CN V; V2, maxillary division of CN V; V3, mandibular division of CN V.

The interdural peeling of the LWCS allowed the visualization of the ACP and the surrounding dura was detached before proceeding with the extradural anterior clinoidectomy. In order to perform this maneuver, the medial portion of the LWS, one of the three anchor points of the ACP, was accurately removed. Then, the orbital roof was taken off, and CN II was identified in the optic canal (OC) (Figure 7A). The clinoidectomy required extreme attention both to avoid damaging the CN II, but also CN III, the superior ophthalmic vein and its branches, ICA, and the proximal segment of the ophthalmic artery. Compared to transcranial extradural clinoidectomy (Figure 8), the visualization of the structures in the OC occurred through an anterior-to-posterior surgical route rather than a lateral one. Considering this perspective, the following structures were recognized, from superior to inferior: the CN II was located supero-medial, the CN III was situated inferolateral and between them, and the paraclinoidal segment of ICA and the proximal segment of ophthalmic artery were found (Figure 7D). Once the two anchor points of the ACP were drilled and the dura was adequately detached, the ACP could be removed by fracturing the optic strut using the forceps (Figure 7B,C).

### 3.2. Anatomo-Radiological Measurements

The analysis of the endocranial surface on the dry skulls highlighted that the average thicknesses of the optic strut and roof were 3.6 ± 0.9 mm (range 2–6 mm) and 1.8 ± 0.6 mm (range 0.9–3.2 mm), respectively. The longitudinal length and transverse diameter of the ACP were 10.2 ± 3.6 (range 5.6–17.4 mm) and 5.5 ± 2.9 mm (range 2.8–8.5 mm). A carotid clinoid foramen between the anterior and middle clinoid processes was identified on five sides (five specimens) (Table 1).

At the level of the orbital region, the thickness of the optic strut and the maximum transverse diameter of the OC were 3.1 ± 1.2 mm (range 1–5 mm) and 7 ± 0.9 mm (range 5–9 mm), respectively. The distances between the OC and the anterior and posterior ethmoid canal were 18.2 ± 3.6 mm (range 8–26 mm) and 6.6 ± 2.5 mm (range 3–16 mm) (Table 1).

**Figure 9:**
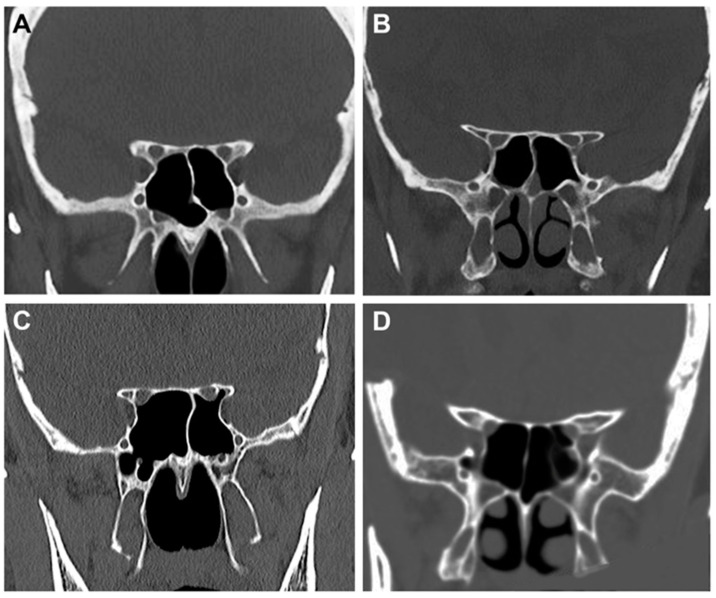
Coronal sections of the 4 variants of the optic nerve identified in the 200 CT scans according to DeLano’s classification [48]. (**A**) Type 1: the optic canal is located laterally to the sphenoid sinus, without any impression. (**B**) Type 2: the right optic canal is characterized by an impression on the lateral wall of the sphenoid sinus. (**C**) Type 3: both optic canals run into the sphenoid sinus. (**D**) Type 4: the right optic canal is characterized by the presence of Onodi cell or spheno-ethmoidal air cell.

The CT measurements (Table 2) were performed on two hundred patients (105 female, 95 male) with a mean age of 68 years (range 21–94 years old). The mean length of the ACP was 11.7 ± 2.4 mm (range 6.4–18.5 mm) with a mean width of 6.4 ± 1.4 mm (range 2.6–9.5 mm). The thickness of the roof of the optic canal and the optic strut were 1.4 ± 0.5 mm (range 0.5–3.5 mm) and 2.5 ± 0.79 mm (range 0.4–5 mm), respectively. The pneumatization of the ACP was identified in 7.5% of cases (15 patients). The relationship between the CN II and the spheno-ethmoidal sinuses was analyzed according to DeLano’s classification [48]: type 1 was identified in 74.5%, type 2 in 17%, type 3 in 7%, and type 4 in 1.5% of patients (Figure 9). The mean work distance between the anterior margin of the orbital cavity floor and the ACP tip was 6.1 ± 0.4 cm (range 5.4–6.8 cm).

Analyzing the clinical cases with the removal of the lateral orbital rim, the working angle on the axial plane increased by 20 ± 5.4° (range 14.7°–27.8°) laterally compared to the trajectory with preservation of the lateral orbital rim.

### 3.3. Clinical Cases

#### 3.3.1. Case 1

**Figure 10:**
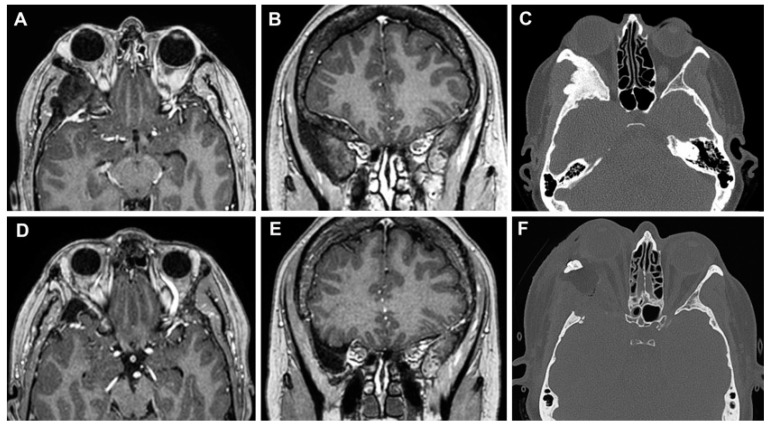
Left sphenoid wing meningioma on preoperative MR images (**A**,**B**) and on axial CT scan (**C**). The post-operative MRI (**D**,**E**) and CT scan (**F**) documented the GTR of the lesion.

A 55-year-old woman presented with a 1-year progressive proptosis in the right eye, associated with occasional episodes of headache and a periorbital, dull, achy sensation. A brain MRI scan documented the presence of a hyperostotic lesion involving the right sphenoid wing and the lateral orbit with adjacent dural thickening, with a mass effect on the lateral rectus muscle (Figure 10A–C). At the pre-operative ophthalmological evaluation, no visual disturbances and cranial nerve deficits were found. A superior eyelid transorbital approach with removal of the lateral orbital rim was performed. The skin incision at the right superior eyelid was started 3 mm lateral to the supraorbital notch. After dissection to the periorbita, a complete exposure of the lateral orbit was obtained. The ACP and the middle fossa were drilled down to V2, although some bone was left to avoid CSF leakage. The dura below the hyperostotic bone was abnormal, and therefore, it was opened and resected. To prevent any CSF leak and infection, the cavity defect was closed with multilayer dural substitutes and fibrin glue, covered by an abdominal fat graft without bony reconstruction. The periosteum and the orbicularis ocularis muscle were reapproximated with a 5-0 absorbable suture in an interrupted fashion, and a running 6-0 fast-absorbing plain gut suture was used for the closure of the skin.

The postoperative course was uncomplicated. Histological analysis confirmed the diagnosis of the sphenoid wing and intraosseous meningioma (WHO grade 1). Five years after surgery, no evidence of bone regrowth or tumor was documented (Figure 10D–F).

#### 3.3.2. Case 2

**Figure 11:**
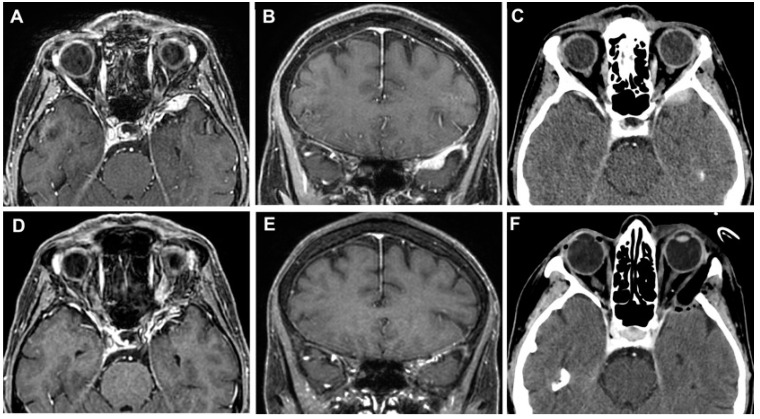
Temporal pole meningioma on preoperative MR images (**A**,**B**) and on axial CT scan (**C**). The post-operative MRI (**D**,**E**) and CT scan (**F**) documented the complete excision of the lesion.

**Figure 12:**
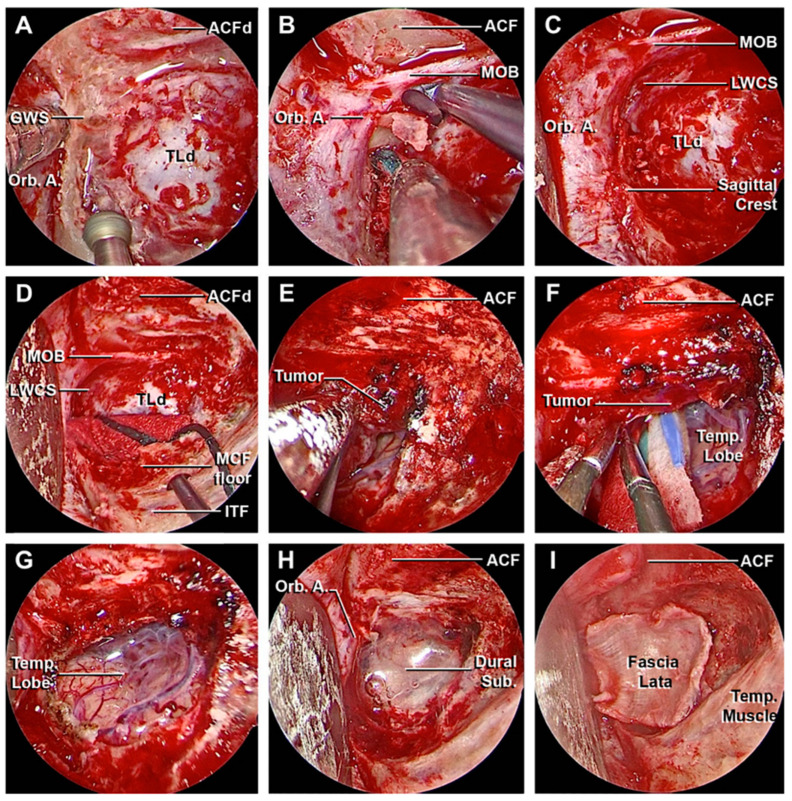
Main steps of the removal of the temporo-polar meningioma. (**A**–**C**) Drilling of the left greater sphenoid wing followed by the exposure of the meningo-orbital band, orbital apex, and the dura mater of the temporal lobe. (**D**–**F**) After opening of the latero-inferior portion of dura mater, the meningioma was progressively dissected from the surface of the temporal lobe. (**G**) Complete excision of the lesion and exposure of the temporal lobe, with the preservation of the arachnoid plane. (**H**,**I**) Accurate hemostasis and plane closure with adipose tissue and fascia lata. ACF, anterior cranial fossa; ACFd, anterior cranial fossa dura; Dural Sub., dural substitute; GWS, greater wing of the sphenoid bone; ITF, infratemporal fossa; LWCS, lateral wall of the cavernous sinus; MCF floor, middle cranial fossa floor; MOB, meningo-orbital band; Orb. A., orbital apex; Temp. Lobe, temporal lobe; Temp. Muscle, temporal muscle; TLd, temporal lobe dura.

A 64-year-old woman presented with a 2-year history of diplopia in the long-distance gaze. A brain MRI scan identified a small extra-axial temporal pole lesion, that continuously increased in subsequent radiological follow-up. (Figure 11A–C). A superior eyelid transorbital approach was performed with an incision along the palpebral crease (Figure 12). After the identification of the lateral orbital rim, the periosteum was incised. Once the superior and inferior orbital fissures were identified, the great sphenoid wing was drilled until the meningo-orbital band and the dura of the temporal pole were completely exposed. The lesion and its dural base were removed, dissecting the portion in contact with the temporal pole following the arachnoidal plane. After thorough hemostasis, a fascia lata graft was used for reconstruction. The postoperative course was characterized by transient diplopia and esotropia of the right eye, which completely resolved three months after surgery. The histological diagnosis was a meningothelial meningioma (WHO Grade 1). The post-operative CT scans and the MRI after 6 months showed the complete removal of the lesion (Figure 11D–F).

#### 3.3.3. Case 3

**Figure 13:**
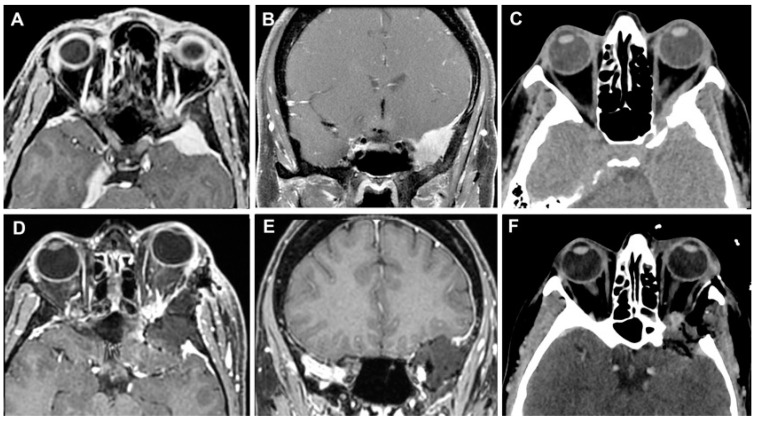
MR images (**A**,**B**) and on axial CT scan (**C**) of a sphenoid wing meningioma. No signs of recurrence were identified in the post-operative MRI (**D**,**E**) and CT scan (**F**).

**Figure 14:**
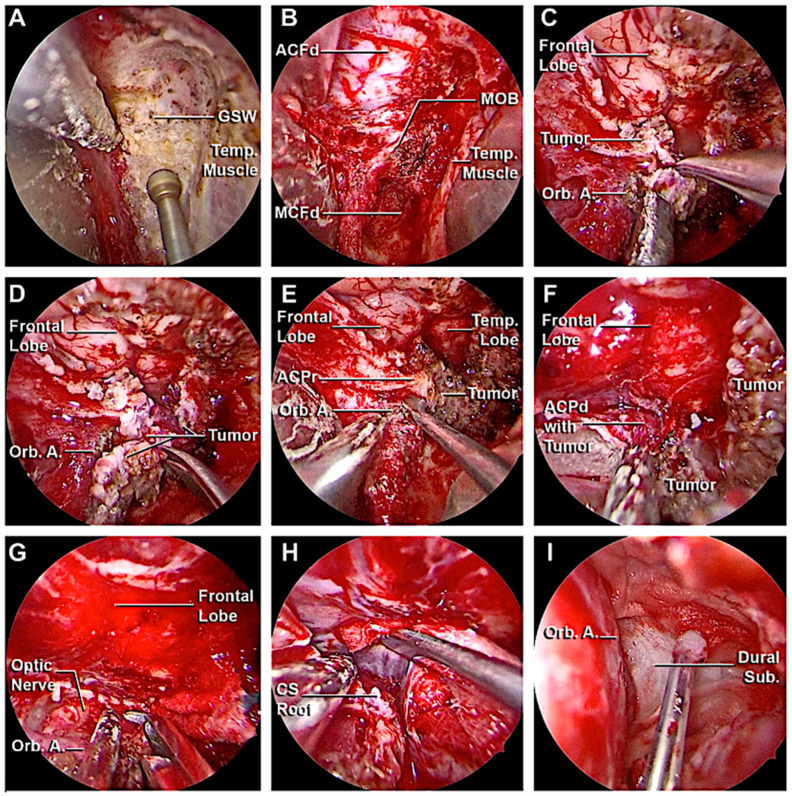
Main steps of the removal of the sphenoid wing meningioma. (**A**,**B**) Drilling of the left greater sphenoid wing with the exposure of the meningo-orbital band and the fronto-temporal dura. (**C**,**D**) Complete removal of the intradural portion of the lesion. (**E**–**H**) Anterior clinoidectomy, with the exposure of the optic nerve and cavernous sinus roof. (**I**) Final hemostasis and closure. ACF, anterior cranial fossa; ACFd, anterior cranial fossa dura; ACPd, anterior clinoid process dura; ACPr, anterior clinoid process root; CS Roof, cavernous sinus roof; Dural Sub., dural substitute; GWS, greater wing of the sphenoid bone; ITF, infratemporal fossa; LWCS, lateral wall of the cavernous sinus; MCF floor, middle cranial fossa floor; MCFd, middle cranial fossa dura; MOB, meningo-orbital band; Orb. A. orbital apex; Temp. Lobe, temporal lobe; Temp. Muscle, temporal muscle.

A 51-year-old woman presented with a 5-year history of progressive pain in the left hemiface, occasionally stimulated by thermal triggers, that was poorly controlled by medical therapy. A hyperostotic lesion on the right side involving the greater and the lesser sphenoid wing with adjacent dural thickening was found in the MRI scan (Figure 13A–C). At the pre-operative ophthalmological evaluation, right-beating nystagmus in the lateral gaze and mild exophthalmos in the right eye were identified. A superior eyelid transorbital approach was performed. The greater sphenoid wing was drilled up to expose the lateral wall of the cavernous sinus and the temporal lobe. An anterior clinoidectomy was also performed due to the tumor involvement of the dura overlying the anterior clinoid process (Figure 14). The dura close to the hyperostotic bone was resected and a fat graft was used to cover the dural defect.

In the immediate post-operative course, the patient reported blurry vision and palpebral motility impairment in the right eye, that progressively improved during the hospitalization. Histological analysis confirmed the diagnosis of secretory meningioma of the sphenoid wing (WHO grade 1). After one month, no signs of recurrence were reported at the radiological follow-up (Figure 13 D–F), with complete regression of the symptoms.

## 4. Discussion

Transorbital approaches represent an innovative surgical route not only for the treatment of orbital pathologies, but also offer a direct surgical corridor to the LWCS [13,20,25,29,40,51,52], and anterior [8,13,28,30,49] and middle cranial fossae [13,19,22,26,27,30,31,32,33,34,35,36,37,38,49,53,54,55,56]. Furthermore, even intraparenchymal targets and specific pathologies can be treated via this route [46,47]. Since TOAs are minimally invasive approaches, in-depth anatomical knowledge is required to recognize the main landmarks despite the unusual surgical perspective, especially for young trainees. This corridor is indicated in the cases of proptosis and lesions located lateral to the CN II [8,13,17,19,20,23,24,31,39,53,54,57]. The TOAs offer a good exposure to the anterolateral skull base through small craniectomies, avoiding excessive brain retraction injury and complications associated with long hospitalization [8,11,12,13,15,17,20,22,23,24,39]. In selected cases, TOAs are valid alternatives to traditional transcranial surgery if the patient is unable to sustain an overly invasive treatment or has comorbidities that would imply greater risks than benefits, allowing for histological diagnosis and the decompression of the main structures involved in the pathology [15,17,22,23,39]. The small surgical incision, hidden by the palpebral skinfold, offers an optimal aesthetic result with minimal impact on the patient’s self-perception [8,20]. Moreover, unlike traditional transcranial approaches, the temporalis muscle is not extensively manipulated, avoiding muscle atrophy [13,20,39].

However, several potential complications must be considered. The palpebral phase must be conducted adequately in order to avoid potential complications related to levator palpebrae muscle injury, such as the risk of post-operative ptosis [13,17,19,20,22,31,53,54,57]. Lateral rectus muscle damage could happen in the case of deep dissection when the intraorbital extension of spheno-orbital meningiomas is found; in this case, other treatment modalities should be applied [38,39,54]. The manipulation, the pressure applied to the eye during surgery, and the damage during the peeling can favor the onset of cranial nerve palsy with associated diplopia or even compressive optic nerve injury [11,16,17,20,22,36,39,52,54,57]. Therefore, it is not uncommon to detect transient deficits in extrinsic ocular motility and visual deficit in the immediate post-operative course, but in most cases, they tend to regress without any consequence on the patient [17,20,22,52,57]. The intermittent release of the retractor and monitoring pupil function can avoid ophthalmic trauma induced by manipulation that occurs during the surgical procedure [13,33,34,49]. The use of wise irrigation reduces the risk of injury caused by heat during the surgical procedure [8,35], but this risk has not been experimentally proven. Obviously, the limited surgical corridor offered by the orbital cavity can be considered, in this context, a risk factor [13,33,34]. Direct or indirect ischemic damages can happen, regardless of the approach. As a matter of fact, most TOAs are periorbita-sparing approaches, at least in the first phases, and vascular damage is possible but not so frequent [17]. Currently, the data from literature obtained from clinical series do not report this event as a common event.

The extradural transorbital anterior clinoidectomy has a surgical trajectory along the longitudinal axis of the ACP, allowing a direct view of the opticocarotid region with a short working distance [35,36,37]. In the transcranial anterior clinoidectomy, a complete lateral view of the middle cranial fossa is obtained at the expense of potential brain retraction injury and a more invasive procedure [58,59]. However, in TOAs, the reduced maneuverability in the cone-pyramid shape space during the opening the orbital roof and clinoidectomy may damage the ICA, ophthalmic artery, and CN III, making the procedure challenging and risky to perform despite the presence of the dura that protects the intradural structures during the maneuvers [35,36,37]. Therefore, in order to perform safe removal of the anterior clinoid process through this surgical corridor, a transcilliar incision could be performed to achieve an approach slightly higher and toward the anterior fossa.

The removal of the lateral orbital rim performed in five clinical cases demonstrated an increase in the working angle on the axial plane by 20 ± 5.4° (range 14.7°–27.8°) laterally with respect to the trajectory with preservation of the lateral orbital rim, improving the surgical maneuverability and especially the resection rate, as described by Kong et al. (54.5.% vs. 26.3% *p* < 0.01) [60]. Recently, Somma et al. analyzed the degree of bony decompression around the CN II through EEA, endoscopic TOA, and transcranial approaches, obtaining the largest degree of circumferential decomposition of the optic canal and the greatest surgical freedom in the latter [61]. Therefore, the extent of the lesion, the patient anatomy, and the goal of surgery determine which approach is the most appropriate.

Before proceeding, a pre-operative thin-slice CT angiography study is suggested to highlight any anatomical variants of the orbital and sellar region, but mostly for eventual ICA anomalies, such as agenesia or hypoplasia. Moreover, the morphological study highlighted the presence of carotico-clinoid foramina in five sides on dry skulls, while in the CT scans, pneumatization of the ACP was found in 7.5% of cases and variants of the course of the optic nerve according to DeLano’s classification were detected [48]. The variability in the thickness and the average length of the main bone structures that must be drilled during the various steps of the surgical approach has been demonstrated. The relationship among the course of the optic nerve, the paranasal sinuses, and the extension of the lesion must be investigated carefully before proceeding with surgical treatment, evaluating the limitations of the TOAs and EEAs.

It should also be kept in mind that several vascular anatomical variants exist. In our study, the meningo-lacrimal artery was recognized in eight cases (80%), while in the literature it was identified in 60% of patients [62]. Although it is frequently closed, in some rare cases, the meningo-lacrimal artery is the main feeder of the central retinal artery. The ophthalmic artery may arise, from example, from the MMA [63]. As well as in the other approaches, the division of the MMA during the extradural peeling at the MCF could inadvertently lead to blindness. Furthermore, the proximal division of the MMA could sacrifice the petrosal branch that supplies the GSPN, resulting in facial nerve palsy and dry eye.

One of the most important limitations to be noted is certainly the limited vision of the operating field and the reduced maneuverability can limit the management of complications and the degree of excision of the lesion. Among the factors that can limit the gross total resection using TOAs are the predominant extension towards the medial region of the cavernous sinus and posterior and infratemporal fossa, multicompartmental tumoral extension, vascularized diseases, and lesion pattern (en-plaque type) [13,17,18,20,22,26,39,52,60,64]. Analyzing different pathologies, most series available in the literature describe a maximal gross total resection (GTR) value from 36.4% to 50% for sphenorbital meningiomas [13,15,16,17,23,26,53,54,60], and 70–81.8% for trigeminal schwannoma, with an average maximum diameter of the lesion around 4 cm when using a subperiosteal tunnel along the orbital in most cases [14,22,64]. The removal of the lateral orbital rim or a combination of the transorbital approach with other surgical approaches increases the GTR rate [21,30,60,65]. Therefore, it is necessary to preoperatively establish the goal of the surgical treatment, evaluating the patient’s comorbidities, the risks and benefits of the surgical procedure, the nature of the lesion, the extension, and the possibility of resorting to adjuvant therapy.

Most of the data available in the literature are preliminary studies on case reports or small groups of patients. Considering the types of lesions commonly treated (spheno-orbital meningiomas and trigeminal schwannomas), the slow tumor growth in most cases, and the novel introduction of this surgical technique, it is difficult to establish the disease recurrence rate due to the scarcity and heterogeneity of available data. Concerning schwannomas, Kong et al., in their clinical series of 50 patients, reported no tumor regrowth or recurrences during 21.9 months of follow-up, although 5 patients had previously undergone treatment (GK or surgery) [64]. Regarding spheno-orbital meningiomas, in their study on 18 patients, In Woo et al. documented tumor progression in 2 patients (11.1%) affected by atypical and transitional meningioma, despite the additional GK therapy [53]. Locatelli et al. reported that one patient, who underwent GTR, presented recurrence after 30 months, while tumor progression was identified after 22.66  ±  15.3 months in three patients who underwent STR [17]. In their study on 22 patients affected by spheno-orbital tumors, Zoli et al. reported a recurrence rate of 31.8% (7 patients) after a mean of 22 ± 12 months [13].

The reconstruction phases are essential to avoid post-operative complications such as CSF leak, infection, meningitis, pseudomeningocele, enophthalmos, chronic subdural hematoma, and subretinal hemorrhage [8,11,13,15,17,20,23,24,52,53,54,56,57]. The use of free fat tissue from the abdomen can be used not only to fill the latero-orbital spaces, but also the space created after removing of the intradural lesion, together with the pressure exerted by the eye. Closure of the dura can be performed either with fascia lata or with dural substitutes combined with dural sealants [13,16,17,19,20,23,26,55,60]. The removed lateral orbital rim segment is replaced in its native position and secured with permanent sutures threaded through pre-drilled holes or plates and screws to protect the globe and maintain a symmetric facial contour.

## 5. Conclusions

The comprehension of the surgical anatomy of the orbit and its surrounding structures is essential to understand the safe application of transorbital approaches. This route is highly suggested in selected cases with tumor extension lateral to the optic nerve, with an average maximum diameter of the lesion around 4 cm, to obtain direct decompression of the optic nerve and main vascular arteries and avoid excessive manipulation of the neurovascular structures.

## Figures and Tables

**Table 1:** Measurements performed on thirty-nine dry skulls (78 sides). ACP, anterior clinoid process; AEC, anterior ethmoidal canal; CC, cranio-caudal; OC, optic canal; PEC, posterior ethmoidal canal.

Measurements on Dry Skulls	
**Endocranial Surface**	
Thickness of Optic Strut	3.6 ± 0.9 mm (range 2–6 mm)
Thickness of Optic Canal Roof	1.8 ± 0.6 mm (range 0.9–3.2 mm)
CC length of ACP	10.2 ± 3.6 mm (range 5.6–17.4 mm)
Transverse Diameter of ACP	5.5 ± 2.9 mm (range 2.8–8.5 mm)
Carotid-Clinoid Foramen	5 sides
**Orbital Region**	
Thickness Optic Strut	3.1 ± 1.2 mm (range 1–5 mm)
Transverse Diameter of OC	7 ± 0.9 mm (range 5–9 mm)
Distance between the OC and AEC	18.2 ± 3.6 mm (range 8–26 mm)
Distance between the OC and PEC	6.6 ± 2.5 mm (range 3–16 mm)

**Table 2:** Data collected from two hundred CT scans. ACP, anterior clinoid process; CC, cranio-caudal; OC, optic canal.

Radiological Measurements	
N° of Patients	200 (105 female, 95 male)
Patient Age Distribution	68 years (range 21–94 ys)
CC Length of ACP	11.7 ± 2.4 mm (range 6.4–18.5 mm)
Width of ACP	6.4 ± 1.4 mm (range 2.6–9.5 mm)
Optic Strut	2.5 ± 0.79 mm (range 0.4–5 mm)
Optic Canal Roof	1.4 ± 0.5 mm (range 0.5–3.5 mm)
Pneumatization of ACP	7.5% (15 patients)
**DeLano’s classification**	
Type 1	74.5% (149 patients)
Type 2	17% (34 patients)
Type 3	7% (14 patients)
Type 4	1.5% (3 patients)

## Data Availability

The authors confirm that the findings of this study are available within the article.
